# Cdx4 and Menin Co-Regulate *Hoxa9* Expression in Hematopoietic Cells

**DOI:** 10.1371/journal.pone.0000047

**Published:** 2006-12-20

**Authors:** Jizhou Yan, Ya-Xiong Chen, Angela Desmond, Albert Silva, Yuqing Yang, Haoren Wang, Xianxin Hua

**Affiliations:** Abramson Family Cancer Research Institute, Department of Cancer Biology, Abramson Cancer Center, University of Pennsylvania, Philadelphia, Pennsylvania, United States of America; Sanofi-Aventis, United States of America

## Abstract

**Background:**

Transcription factor Cdx4 and transcriptional coregulator menin are essential for *Hoxa9* expression and normal hematopoiesis. However, the precise mechanism underlying *Hoxa9* regulation is not clear.

**Methods and Findings:**

Here, we show that the expression level of *Hoxa9* is correlated with the location of increased trimethylated histone 3 lysine 4 (H3K4M3). The active and repressive histone modifications co-exist along the *Hoxa9* regulatory region. We further demonstrate that both Cdx4 and menin bind to the same regulatory region at the *Hoxa9* locus in vivo, and co-activate the reporter gene driven by the *Hoxa9 cis*-elements that contain Cdx4 binding sites. Ablation of menin abrogates Cdx4 access to the chromatin target and significantly reduces both active and repressive histone H3 modifications in the *Hoxa9* locus.

**Conclusion:**

These results suggest a functional link among Cdx4, menin and histone modifications in *Hoxa9* regulation in hematopoietic cells.

## Introduction

Homeo-box-containing transcription factors (Hox proteins) play a pivotal role in normal differentiation and expansion of hematopoietic cells [1], [2]. Overexpression of several *Hox* genes such as *Hoxa9* induce leukemia reminiscent of mixed lineage leukemia (MLL) [1]. The *MLL* gene is frequently targeted by chromosomal translocations in acute lymphoid and myeloid leukemias (ALL and AML, respectively) [3], resulting in generation of various MLL-fusion proteins with the amino portion of MLL fused in frame with one of over 50 different potential fusion partners. Many MLL fusion proteins cause dysregulation of Hox genes including *Hoxa9*. Both human and murine leukemias triggered by MLL-AF9 are most frequently myeloid in phenotype [4]–[6].

Menin was originally identified as a tumor suppressor encoded by *Men1* that is mutated in the human inherited tumor syndrome, multiple endocrine neoplasia type 1 (*MEN1*) [7], [8]. It interacts with a variety of transcriptional factors and MLL proteins [9]–[12]. Recently, we and others have shown that menin regulates hematopoiesis and myeloid transformation by influencing *Hox* gene expression [13], [14]. Menin and MLL fusion proteins are required for persistent expression of 5′ Hoxa cluster genes, including *Hoxa5* to *Hoxa10* [14]. Despite the advances in understanding the oncogenic contributions of the menin-MLL complex, precisely how expression of Hoxa cluster genes is regulated in hematopoietic cells remains unclear.

The vertebrate *Cdx* genes (*Cdx1*, *Cdx2* and *Cdx4* in the mouse) encode homeodomain transcription factors related to the *Drosophila* caudal genes. They belong to the ParaHox gene family and share common ancestry with the *Hox* genes [15]. In zebrafish, a *Cdx4* mutant, *kgg^tv205^*, causes an early defect in hematopoiesis and aberrant expression of multiple *Hox* genes, particularly *Hoxb6b* and *Hoxa9a* [16]. Ectopic expression of Cdx4 induced a pronounced expansion of multi-lineage progenitors from differentiating mouse embryonic stem cells, including an increase in granulocyte-erythroid-macrophage-megakaryocyte colonies and in granulocyte-macrophage colonies, as compared with control cells [16]. These data indicate that Cdx4 is essential for *Hoxa9* regulation in normal hematopoiesis.

There is substantial data demonstrating an essential role for Hoxa9 protein in both normal hematopoiesis [17] and leukemic transformation induced by certain MLL fusion proteins [18]. *Hoxa9* overexpression is a common feature of acute myeloid leukemia [19]–[21]. Although both menin and Cdx4 have been shown to participate in regulating *Hoxa9* gene transcription and hematopoiesis, little is known as to whether or how Cdx4 interacts with the *Hoxa9* locus to regulate its expression, and whether menin and Cdx4 crosstalk to regulate expression of Hoxa cluster genes.

In this study, we used two MLL-AF9 transformed cell lines, AT1 and AR1, as a model system to investigate *Hoxa9* regulation. We showed that histone modifications, especially methylation of histone H3, play an important role in determining the level of *Hoxa9* expression, and menin is crucial for both active and repressive histone modifications in the *Hoxa9* locus. We also demonstrated a crosstalk between menin and Cdx4 in co-regulating expression of *Hoxa9* gene. These results uncovered a functional link among Cdx4, menin and histone modifications of histone H3 in maintaining 5′ Hoxa cluster transcription, thus providing novel insights into our understanding of regulation of *Hox* gene expression.

## Results

### Distinct levels of *Hox* gene expression in two MLL-AF9 transformed cell lines

**Figure 1:**
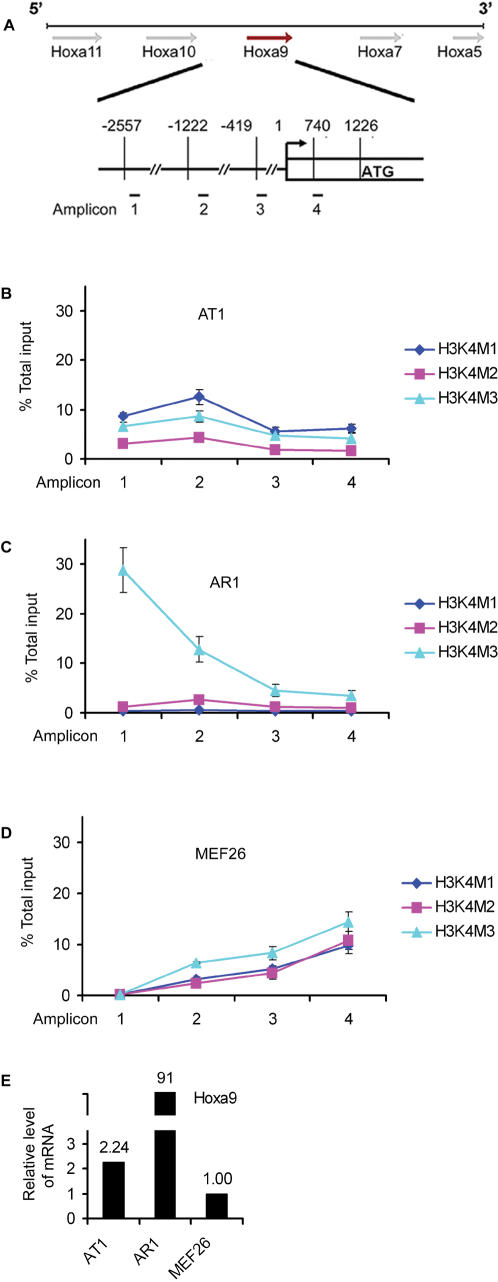
Location of H3K4M3 correlated with *Hoxa9* expression level. (A) Diagram of 5′ Hoxa cluster genes, from *Hoxa5* to *Hoxa11*, showing the location of the amplicons used for quantitative Chromatin Immunoprecipiation assay (qChIP). The enrichment of H3K4 methylation was determined by qChIP in AT1 cells (B), AR1 cells (C) and MEF26 cells (D). (E) *Hoxa9* expression levels in the indicated cell lines as determined by qPCR. The relative expression level of *Hoxa9* gene was calculated relative to that in MEF26 cells.

AT1 and AR1 cell lines were both transformed by transduction of *MLL-AF9* fusion genes, however, compared to AT1, AR1 cells were cultured easily and grew faster (S Fig. 1A). The expression levels of *Hoxa5*, *Hoxa7* and *Hoxa9* in AR1 cells, as measured by qPCR, were 20 to 40 fold higher than those in AT1 cells. The *Hoxa10* level in AR1 cells was even 105 fold higher than in AT1 cells (S Fig. 1B), while the expression of *Hoxa1* and *Hoxa13* was not detectable in AT1 cells. These results suggest that expression of multiple *Hox* genes in AR1 cells is generally higher than that in AT1 cells, consistent with a more aggressive phenotype of AR1 cells (S Fig. 1C).

More importantly, the morphology and expression profile of 5′ Hoxa cluster genes in AT1 and AR1 cells are strikingly similar to the previously published two distinct stages of MLL-AF9 transformed hematopoietic cells: preleukemic and leukemic stages [4], [22], [23]. In those studies, the authors found that MLL-AF9 fusion induced generation of preleukemic cells followed by overt leukemic cells [4]. In contrast to leukemic cells, preleukemic cells possessed a high differentiation potential and expressed a lower level of 5′ *Hoxa* cluster genes [22], [23]. Mouse embryonic fibroblast (MEF) cells were often used to study histone modifications in *Hoxa9* gene expression [24], [25]. The expression level of *Hoxa9* in AT1 cells was only two fold higher than in MEF cells (Fig. 1E). Apparently AT1 cells have not transformed into overt leukemic cells but possessed differentiation potential associated with an early stage of MLL-AF9 transformation, similar to the preleukemic cells [4]. Therefore, utilization of AR1 and AT1 cell lines would facilitate our understanding of the altered regulation of *Hox* genes in various stages of leukemia.

### Multiple functional elements spread over the regulatory sequence of *Hoxa9* gene

**Figure 2:**
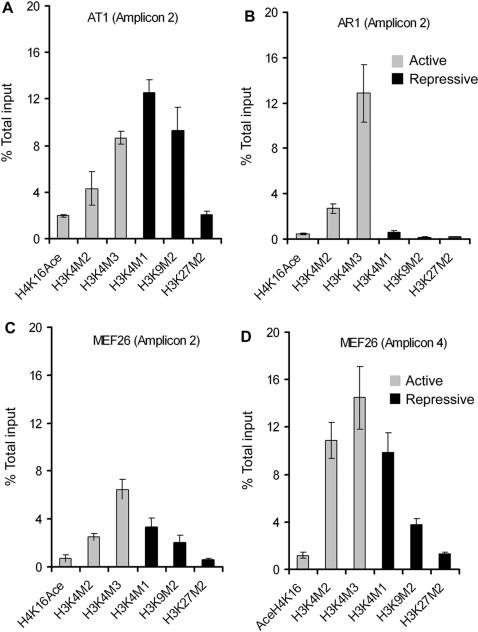
Active and repressive histone modification markers were co-enriched in *Hoxa9* locus. Active histone markers including H4K16Ace, H3K4M2 and H3K4M3, were indicated as grey bars. Repressive markers including H3K4M1, H3K9M2 and H3K27M2 were indicated as black bars. (A) AT1 cells maintain both active and repressive histone modifications at amplicon 2. (B) AR1 cells show aberrant high expression of active histone modifications but low level of repressive histone modifications at amplicon 2. (C) and (D) show that MEF26 cells contain both active and repressive histone markers at amplicons 2 and 4, respectively.

Initiation of *Hox* gene transcription requires interaction among numerous factors recruited to the target gene, particularly to the multiple functional elements, so as to establish their precise domain boundaries in the developing tissue [26]. The *Hoxa9* regulatory region may serve as binding sites for various transcription factors and co-regulators to regulate the magnitude of *Hoxa9* expression. To identify the regulatory elements in the *Hoxa9* gene, we employed a luciferase reporter assay to quantitatively analyze the cis-elements in the *Hoxa9* locus to regulate its expression. Various cis-elements ranging from −1222 to +1226 of the *Hoxa9* locus were inserted upstream of the luciferase reporter to generate reporters pA through pF (S Fig. 2A). The effects of each regulatory element on reporter gene activity were tested in MEFs (S Fig. 2B). Luciferase assays identified a strong activating element located at 5′ promoter region, B, and two other activating elements within the 5′ untranslated region (5′-UTR), E and F. A repressive fragment, as represented by elements C and D, is located between the two activating elements, B and E (S Fig. 2B). These results suggest that both positive and negative regulators are involved in regulating *Hoxa9* transcription through distinct cis-elements in the *Hoxa9* locus.

### The level of *Hoxa9* expression correlates with the location of the increased lysine 4-trimethylated histone H3 in the *Hoxa9* locus

We and others have separately demonstrated that the menin and MLL complex [13], [14] and the SUZ12-EZH2 (polycomb proteins) [27] interact with *Hoxa9* regulatory regions. MLL confers histone H3 lysine 4 (H3K4) methlyation, while SUZ12-EZH2 mediates H3K27 methylation [28], [29]. However, precisely how histone H3 modifications are distributed among positive and negative elements in the *Hoxa9* locus is poorly understood. Having identified the potentially active and repressive element, we used quantitative chromatin immunoprecipitation (qChIP) to map a variety of histone modifications in the *Hoxa9* locus, as represented by amplicons 1–4 (Fig. 1A) in three different cell lines, each expressing a distinct level of *Hoxa9* (Fig. 1E).

The patterns of H3K4 methylation across the regulatory region of *Hoxa9* are distinct among the three cell lines (Fig. 1B to D). In AT1 cells, which express a medium level of *Hoxa9* among the three cell lines, there were marked enrichments of multiple histone modification markers including H3K4 trimethylation (H3K4M3), H3K4 dimethylation (H3K4M2) and H3K4 monomethylation (H3K4M1) that peaked at amplicon 2 and extended across amplicons 1, 3 and 4. The enriched level of H3K4M3, H3K4M2 and H3K4M1 accounted for 4–9%, 2–4% and 6–13% of the input among the four regions, respectively (Fig. 1B). In contrast, a different pattern of H3K4 modifications was detected in AR1 cells, which express a much higher level of *Hoxa9*, 41 fold higher than in AT1 cells (Fig. 1E). Although the level of H3K4M3 at amplicons 3 and 4 was comparable to that in AT1 cells, it gradually increased toward 5′ amplicons 1–2, culminating at amplicon 1 to 28% of the input in AR1 cells, as compared to 6.5% in AT1 cells (Fig. 1C). On the other hand, H3K4M1 and H3K4M2 in AR1 cells were either quite low or barely detectable. To compare our results of histone modifications with previously published data with MEFs [24], [29], we also measured H3K4 modifications among the 4 amplicions in MEFs which express a low level of *Hoxa9* (Fig. 1E). In contrast to AR1 cells, no H3K4 methylations were detected at amplicon 1, while H3K4M3 was gradually increased from 6% to 14% from amplicons 2 to 4. Enrichment of H3K4M1 and H3K4M2 mimicked the trend of H3K4M3, albeit at a relatively lower level. Although H3K4 methylations in MEF26 cells were maintained at a relatively high level at amplicon 4, the expression of *Hoxa9* gene was low (Fig. 1E). These results demonstrate that H3K4 trimethylation at the 5′ sequence examined in the *Hoxa9* locus is closely correlated with a high level of *Hoxa9* expression, suggesting the importance of the location of methylated H3K4 in upregulating *Hoxa9* expression.

### Active and repressive histone modifications coexist in the *Hoxa9* regulatory region

It has been reported that H3K4M2 and H3K4M3, and acetylation of histone 4 lysine 16 (H4K16Ace) are correlated with transcriptionally active genes, whereas H3K4M1, dimethylation of histone 3 lysine 9 (H3K9M2) and dimethylation of histone 3 lysine 27 (H3K27M2) are associated with transcriptional silencing [30]–[32]. Although methylation of histone H3K4, H3K9 and H3K27 are important for maintenance of normal *Hoxa9* expression [27], [28], [33], [34] whether these positive and repressive histone modifications co-exist in *Hoxa9* regulation is unclear.

As noticed in AT1 cells, both active (H3K4M2, H3K4M3 and H4K16Ace) and repressive (H3K4M1, H3K9M2 and H3K27M2) histone modification markers were differentially enriched at the 5′ promoter region (amplicon 2) (Fig. 2A). In contrast, in AR1 cells H3K4M3 was highly increased but other modifications were suppressed (Fig. 2B). Similar to AT1 cells, MEF26 cells possessed both active and repressive histone modifications at both amplicons 2 and 4 (Fig. 2C, D). Enrichment of both active and repressive chromatin at the same region suggests that they may antagonize or cooperate to establish a dynamic balance. This balance could be useful for maintaining the level of *Hoxa9* expression. An alteration in this balance could result in deregulation of *Hoxa9* expression. This is consistent with hyperexpression of *Hoxa9* in AR1 cells in which the repressive histone modifications in the *Hoxa9* locus markedly reduced (Fig. 1C, 1E and 2B). It is likely that the co- enrichment of repressive histones moderates the high expression of *Hoxa9*.

### Menin is required for active and repressive histone H3 modifications in the *Hoxa9* locus and the expression of 5′ Hoxa cluster genes

**Figure 3:**
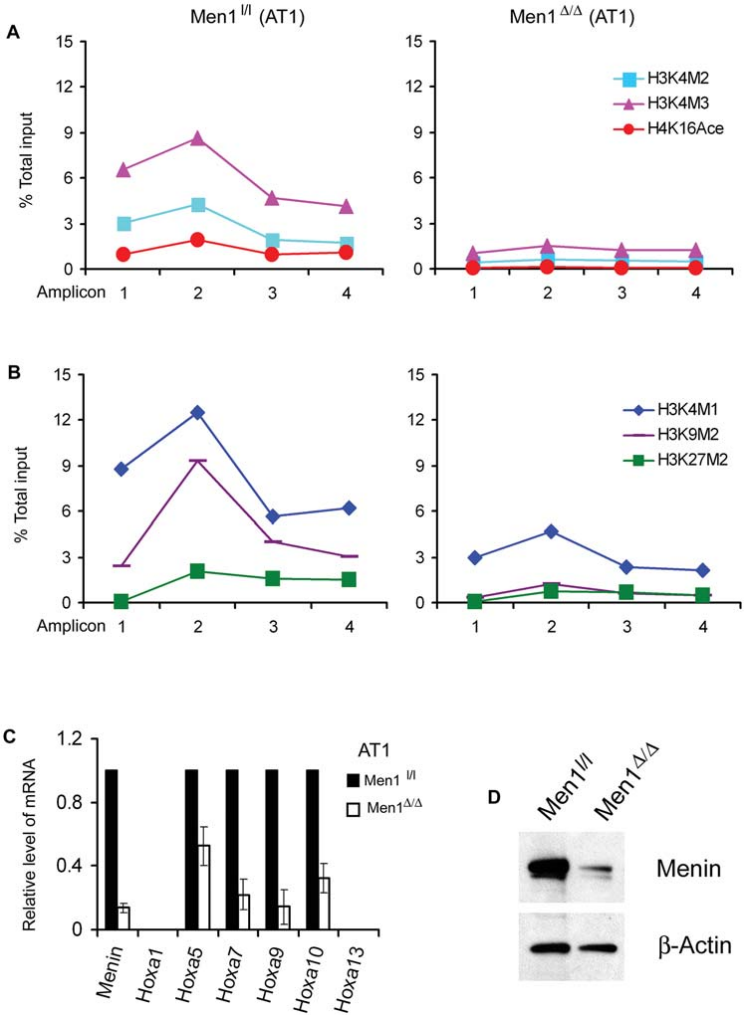
Menin is required for both active and repressive histone modifications in AT1 cells. Menin excision (*Men1^Δ/Δ^*) dramatically reduced active (A) and repressive (B) histone modifications. The highly enriched peak of H3K4M3 and H3K4M2 were almost completely abrogated. (C) Menin excision also reduced the expressions of *Hoxa5*, *Hoxa7*, *Hoxa9* and *Hoxa10*. No expression of *Hoxa1* and *Hoxa13* was detectable. (D) 4-OH Tamoxifen (4-OHT) treatment substantially reduced menin expression as compared to non-tamoxifen treated AT1 cells. Mouse β-actin was used as control.

To evaluate the role of menin in histone modifications at various locations of the *Hoxa9* locus, we treated AT1 cells with either control DMSO or 4-OH tamoxifen (4OHT) to excise floxed *Men1*, followed by qChIP assay. 4OHT markedly reduced menin expression (Fig. 3D). In menin-expressing AT1 cells, H3K4M3 associated with each of the 4 amplicons, reaching 6.5–9% of the input at amplicons 1–2 (Fig. 3A, right panel). Other histone modifications, H3K4M2 and H4K16Ace, were also detectable at amplicons 1–4, albeit at a lower level (Fig. 3A, right panel). In contrast, *Men1* excision (*Men1* ^Δ/Δ^) markedly reduced all three active histone modifications (Fig. 3A, right panel). This result is in agreement with the previous observation that menin facilitates trimethylation of H3K4, activates *Hoxa9* expression [13], and also links menin's function to H4K16Ace. Notably, *Men1* excision also markedly reduced all three repressive histone modifications at the 4 amplicons of the *Hoxa9* locus (Fig. 3B).

To verify the functional links between menin-associated histone 3 modifications and *Hox* gene expression, we conditionally excised menin in AT1 cells and examined menin's influence on the expression of 5′ Hoxa cluster. In keeping with reported results that *Hoxa5* –*a10* are co-expressed in MLL-AF9 transformed cells [14], [18], [23], menin excision significantly reduced the expression of *Hoxa9* (7 fold), *Hoxa7* (5 fold), *Hoxa5* (2 fold) and *Hoxa10* (3 fold) (Fig. 3C). These results suggest that menin is required to maintain the expression of 5′ Hoxa cluster genes in hematopoietic cells through mediating multiple methylations at histone 3.

### Cdx4 is required to regulate the expression of 5′ Hoxa cluster genes at a certain stage of hematopoiesis

**Figure 4:**
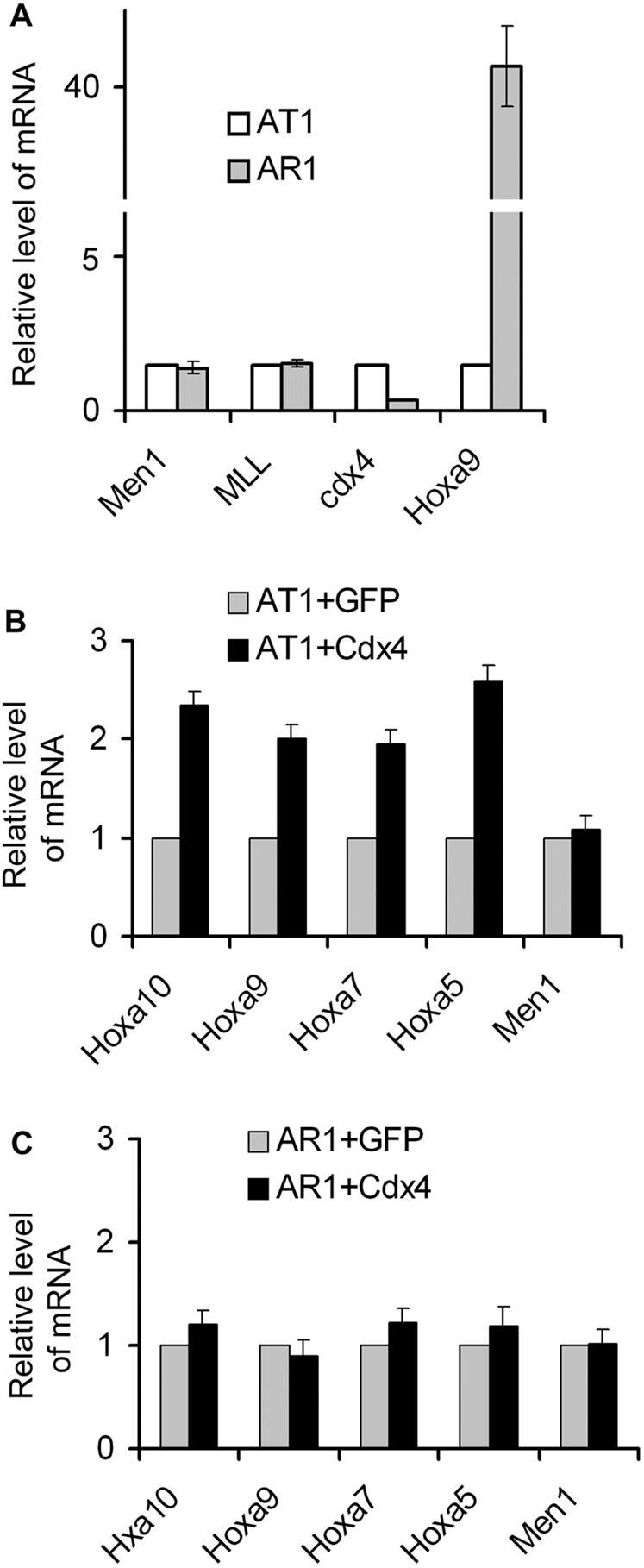
Cdx4 is crucial for the expression of 5′ Hoxa cluster genes in hematopoietic AT1 cells. (A) Expression profile of *Men1*, *MLL*, *Cdx4* and *Hoxa9* was determined by quantitative real time RT-PCR. Ectopic expression of Cdx4 increased the expression of 5′ Hoxa cluster in AT1 cells (B) but not in AR1 cells (C).

Previous studies have shown that menin and Cdx4 induce hematopoiesis at least in part through regulating *Hox* genes, especially *Hoxa9* [13], [16]. In agreement with the finding that Cdx4 is required for *Hoxa9* and *Hoxa7* expression [16], our retrovirus-based *Cdx4* shRNAs experiments showed that *Cdx4* knockdown decreased the expression of 5′*Hoxa* cluster genes, *Hoxa5*- *a10*; particularly *Hoxa9* expression was reduced by 38 fold relative to the control (data not shown). These data suggested that the *Cdx4* gene is crucial for 5′Hoxa cluster expression in AT1 cells, and especially for *Hoxa9* regulation. As Cdx4 is almost absent in AR1 cells (Fig. 4A), we infected AT1 and AR1 cells by Cdx4-expressing retrovirus. The same vector containing IRES-GFP but not Cdx4 cDNA was used as control. We noticed that ectopic expression of *Cdx4* increased the expression of Hoxa cluster genes 2 fold in AT1 cells (Fig. 4B) but did not change the expression of these genes in AR1 cells (Fig. 4C). Menin expression was not influenced by expression level of Cdx4 in both AT1 cells and AR1 cells (Fig. 4 B, C). Given that AT1 cells express a lower level of Hoxa genes and have the potential to differentiate into myelomonocytes and myeloneutrophils (S Fig. 1C), we concluded that *Cdx4* gene may be temporarily required to establish and modulate *Hox* gene expression at specific stages of hematopoiesis, possibly during myeloid transformation [16], [35]. However, Cdx4 is no longer required once Hoxa cluster genes have been highly activated in cells such as AR1 cells. Consistent with this notion, in AR1 cells *Hoxa9* expression was 41 fold higher than that in AT1 cells but *Cdx4* expression was almost absent (Fig. 4A).

### Cdx4 and Menin co-regulate expression of the reporter activity driven by *Hoxa9* cis-elements

**Figure 5:**
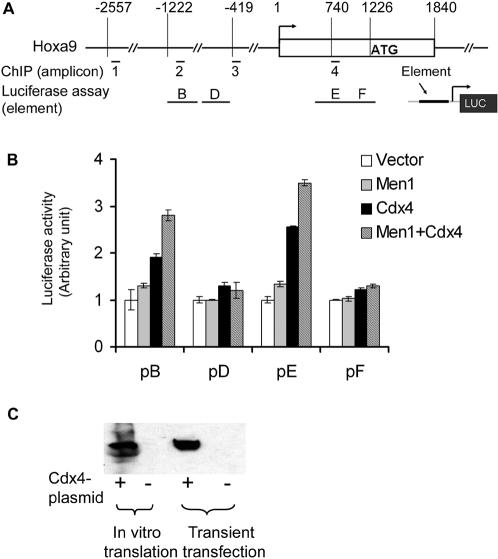
Cdx4 and menin co-regulate the reporter activity driven by *Hoxa9* regulatory elements. (A) Diagram of *Hoxa9* locus, showing the locations of amplicons and the corresponding reporter elements B, D, E and F. (B) Co-expression of menin and Cdx4 with reporters in 293 cells induced the activity of reporters pB and pE. The reporter activity was arbitrarily calculated relative to vector control. (C) Western blot shows that *in vitro* synthesized and transiently expressed Cdx4 was specifically detected by anti-Cdx4 antibodies.

We further determined whether Cdx4 cooperates with menin in *Hox* expression. To this end, we used *Hoxa9* gene as a model to evaluate the effect of menin and Cdx4 on reporter gene activity (Fig. 5A). We cotransfected four reporters (pB, pD, pE and pF) containing different *Hoxa9* regulatory elements with menin and/or Cdx4-expressing constructs into 293 cells. As shown in Fig.5B, when reporter pB was cotransfected with Cdx4 and/or menin, its activity was induced by Cdx4 and enhanced by co-expressed menin (Fig. 5B). Neither menin nor Cdx4 alone had significant effects on reporters pD and pF (Fig. 5B). Interestingly, cotransfecting both menin and Cdx4 with reporter pE yielded an additive activation of the reporter expression by 3.5 fold higher than either menin (1.3 fold) or Cdx4 (2.5 fold) alone (Fig. 5B). Thus, the luciferase assays suggest that Cdx4 could directly activate the activity of the *Hoxa9* elements, and menin may enhance its activation.

### Cdx4 protein specifically binds to two sites in the *Hoxa9* regulatory region *in vitro*

**Figure 6:**
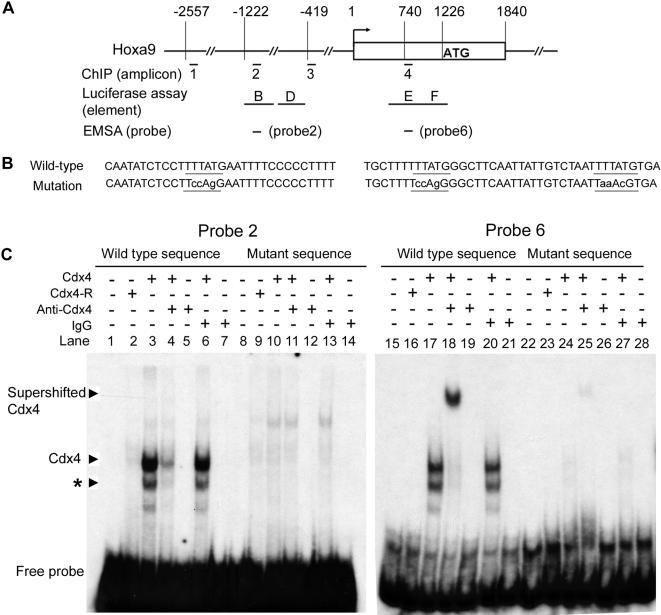
*In vitro* detection of Cdx4 protein binding sites in the regulatory region of *Hoxa9*. (A) A diagram of *Hoxa9* locus, showing the corresponding locations between amplicons for qChIPs, reporter elements for luciferase analysis and oligo probes for EMSA. (B) The wild-type and mutation sequences of probe 2 (left) and probe 6 (right). The Cdx protein consensus binding sites were underlined. Wild type sequence of Probe 2 contains one potential Cdx consensus binding site (CBSs) while probe 6 contains two tandem CBSs. (C) Cdx4 protein was able to bind probe 2 wild type sequence but not the mutant sequence. Asterisk (*) denotes a band that likely resulted from partially degraded Cdx4. Their binding was inhibited by anti-Cdx4 antibody but not by a control IgG (left panel). The same *in vitro* binding assay was conducted for probe 6 and its mutant (right panel).

To determine whether Cdx4 directly interacts with *Hoxa9* regulatory elements, we performed sequence homolog search for the consensus binding sites (TTTATA/G) for Cdx4 [36]. The search revealed three Cdx4 binding sites (CBS): one in the 5′ part of element B and the other two closely located within element E (Fig. 6A and B). We then performed gelshift assays to establish whether Cdx4 can bind these sites. Of six wild type oligonucleotide sequences used as probes (see material and methods), only two probes (probe 2 and 6) were specifically bound by *in vitro* synthesized Cdx4 protein (A.S. data not shown). We then focused on these two probes. Both probes 2 and 6 bound the *in vitro* translated Cdx4 (Fig. 6C, lanes 3 and 17), but not the control lysate (lanes 2 and 16). Moreover, Cdx4/probe 2 complex was abolished by incubation with an anti-Cdx4 antibody but not by control IgG (lanes 4 and 6), in agreement with the previous report [36], suggesting that Cdx4/probe 2 interaction or stability of the complex was diminished by the antibody. However, Cdx4/probe 6 complex was further supershifted by the same anti-Cdx4 antibody, but not by the control IgG (Fig. 6C, lanes 18 and 20). Since probe 6 contains two CBS tandem repeats with surrounding nucleotide sequences different from probe 2, probe 6 might form a more stable complex with Cdx4 protein. Similar to most homeodomain proteins, Cdx4 possibly forms a dimer to bind to target DNA [37]–[39]. Thus, two tandem sites may stabilize the Cdx4/ DNA interaction, resulting in supershift of Cdx4/DNA complex.

To further evaluate the specificity of Cdx4 and probes 2 and 6 interaction, we introduced three nucleotide substitutions into the CBSs in each probe (Fig. 6B), and each of the mutated probes failed to bind Cdx4 (Fig. 6C, lanes 8–14, and 22–28). This series of experiments indicate that Cdx4 interacts *in vitro* with CBSs from element B and E (Fig. 5A), which confers Cdx4 direct upregulation to reporter expression.

### Menin modulates Cdx4 access to the chromatin targets in *Hoxa9* locus

**Figure 7:**
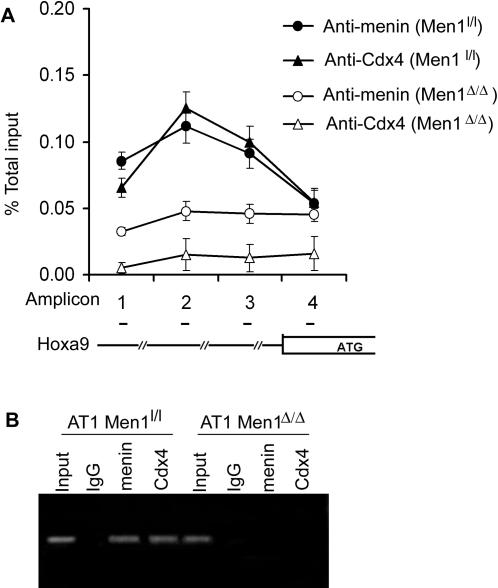
Loss of menin abolishes Cdx4 binding to chromatin targets. (A) qChIP assay shows that Cdx4 and menin have a similar binding peak at amplicon 2. Menin excision (AT1 *Men1^Δ/Δ^*) almost completely blocked Cdx4 binding. (B) The amplified PCR product for amplicon 2 was separated on 1.5% agarose gel.

To determine whether Cdx4 associates with the *Hoxa9* locus *in vivo*, we performed qChIP assays. We found that menin and Cdx4 co-localized to the 5′ promoter region, and particularly enriched a region represented by amplicon 2 (Fig. 7A). Menin ablation abolished Cdx4-chromatin binding, while menin binding to the region was also markedly reduced due to menin excision from the cells (Fig.7A). Agarose electrophoresis of PCR products confirmed that the appropriate target was amplified (Fig. 7B). Unexpectedly, amplicon 4, which contains two CBSs was not as efficiently bound by Cdx4 as amplicon 2 (Fig. 7A). Thus, Cdx4 binding to the chromatin target depends on accessibility of the targets, and menin facilitates association of Cdx4 with the *Hoxa9* locus.

## Discussion

*Hox* genes play an essential role in controlling normal hematopoiesis as well as leukemogenesis [1], [40]. The expression of *Hox* gene is tightly regulated during the process of hematopoietic proliferation and differentiation. For example, *Hoxa9 and Hoxa7* are expressed in primitive progenitors and downregulated at the time of differentiation [40]–[42]. Histone modifications provide a dynamic and versatile code for the recruitment of factors that control gene transcription [43]. We are the first to have demonstrated that Cdx4 directly bound the *Hoxa9* locus and cooperated with menin to regulate *Hoxa9* expression. Thus, our results reveal two distinct yet related mechanisms in regulating *Hoxa9* expression in hematopoietic cells: Cdx4 binding to the CBSs and menin-dependent multiple H3K4 methylations at the *Hoxa9* locus.

### Menin-dependent H3K4 methylation is closely correlated with expression of the 5′ Hoxa cluster in MLL-AF9 transformed cells

Modern tumor biology has emphasized a quantitative model by which cells undergo gradual ‘progressive’ alterations, taking them from normal phenotype to abnormal (precancer) and to malignant phenotype (cancer) [44], [45]. To this end, we applied a quantitative strategy to link the expression level of *Hox* genes with the enrichment of specific histone methylations in *Hoxa9* regulatory region. This was performed in several cell lines with distinct levels of *Hoxa9* expression in decreasing magnitude: AR1, AT1, and MEF26 cells. In correlation with an increase of *Hoxa9* expression, an enriched peak of H3K4M3 was shifted from 3′ to 5′ of the *Hoxa9* regulatory sequence from MEF26 to AR1 (Fig. 1). Trimethylated H3K4 has been reported to associate primarily with the promoter regions of actively expressed genes [46], [47] and extended across the *Hoxa9* locus in MLL-fusion transformed cells [46], [48]. Moreover, our data suggest that the extension of trimethylated H3K4 towards the 5′ *Hoxa9* regulatory region is closely correlated with increasing expression of *Hoxa9*. It is possible that H3K4M3 in the 5′ part of the locus is more efficient in recruiting cofactors, such as chd1 which activates gene transcription by chromatin remodeling to stimulate gene transcription [9], [13], [49], [50]. Further studies are required to clarify this possibility. Despite loss of the SET domain in MLL fusion proteins, they can actively increase binding of the endogenous and intact MLL to the target genes 5 to 15 fold, leading to increased H3K4 trimethylation [48]. Thus, one way that menin regulates expression of *Hoxa9* and other Hoxa cluster genes may be to modulate the recruitment of endogenous MLL and MLL-AF9 to a particular locus to alter histone modifications.

Detection of the co-existence of active and repressive histone modifications at the *Hoxa9* locus is consistent with the notion that certain genes controlling cell “stemness” or plurotency are coordinately regulated by active and repressive chromatin modifications [46], [51]. Alternations of this co-existence may also influence the expression level of *Hoxa9*. For instance, in AT1 cells and MEF26 cells both active and repressive histone modification markers were enriched whereas, repressive histone modifications were significantly diminished in AR1 cells that express a high level of *Hoxa9*.

We further demonstrated that menin is a crucial regulator for both active and repressive histone 3 modifications in the *Hoxa9* locus. Ablation of menin almost abolished both active and repressive histone modifications and reduced expression of 5′Hoxa cluster genes, *Hoxa5* to *a10* in AT1 cells. Menin associates with MLL and the *Hoxa9* locus [13], [14], [29], [52]. Moreover, our new findings suggest the possibility that menin may play a role in the communication between activator and repressor factors in *Hox* gene regulation. Further studies are needed to decipher this function.

### Cdx4 binds the *Hoxa9* locus and is required for *Hoxa 9* expression in normal hematopoietic cells

Cdx4 is required for normal hematopoiesis, stem cell function, and *Hoxa9* expression in both mice and Zebrafish [16], [35]. Enforcing its crucial role, a point mutation of Zebrafish Cdx4 homolog abrogates normal hematopoiesis and *Hoxa9* transcription, while ectopic expression of *Hoxa9* can partially rescue the defect [16]. However, it remains unclear whether Cdx4 directly binds any of *Hox* genes' loci to regulate their transcription. We have now shown that Cdx4 directly binds to the CBSs in the 5′ portion of the *Hoxa9* locus, both *in vitro* and *in vivo*. Significantly, knockdown of Cdx4 led to marked reduction of multiple 5′ Hoxa cluster genes, including *Hoxa9* in AT1 cells (Fig. 4B), whereas ectopic expression of Cdx4 upregulates expression of multiple endogenous Hoxa cluster genes including *Hoxa9*. These results indicate an essential role for Cdx4 in promoting expression of endogenous *Hoxa* genes. Further supporting these observations, Cdx4 upregulates expression of the reporter driven by the *Hoxa9* elements containing CBSs, but not the reporter driven by elements without CBSs (Fig. 5). Together, these results indicate that Cdx4 can directly bind the *Hoxa9* locus to promote transcription of *Hoxa9*, uncovering a direct link between Cdx4 and expression of *Hox* genes.

### Cdx4 and menin co-regulate the expression of 5 Hoxa cluster genes

We have shown that Cdx4 and menin bound to the same region in the *Hoxa9* locus, and they also additively activate the cis-element from the locus. Importantly, loss of menin prevents Cdx4 targeting the *Hoxa9* gene. The precise nature of the interaction among menin, Cdx4 and chromatin DNA targets is not yet clear, but could involve the menin-MLL complex, which is known to mediate H3K4 methylation. However, we do not have evidence that Cdx4 directly interacts with menin in co-immunoprecipitation assay (A.S., data not shown). Histone modifications can directly affect chromatin structure and present a special surface for interaction with other proteins, resulting in either activation or repression of gene transcription [43]. Although Cdx4 possesses intrinsic ability to bind the CBSs, Cdx4 itself is not sufficient to initiate *Hoxa9* expression in the embryonic stem cells (data not shown). Menin-dependent histone modifications via MLL in the *Hoxa9* locus may further recruit/stabilize Cdx4 in the chromatin and thus, establish an optimal level of Hoxa cluster gene expression at certain developmental stage of hematopoietic cells, such as AT1 cells. Obviously, menin is an essential oncogenic cofactor to regulate *Hoxa9* expression in MLL-associated leukemogenesis [14] whereas Cdx4 is no longer required to maintain a high level of *Hox* gene expression in AR1 cells (Fig. 4B, 4D). Thus, Cdx4 may function as a temporal regulator in establishment and/or modulation of *Hox* expression. Indeed, Cdx proteins have long been recognized as playing an important role in the initiation of *Hox* gene expression [26] in a dosage-dependent mode [53].

### Aberrant histone modifications are implicated in MLL-associated leukemogensis

MLL-AF9 promotes expansion of myeloid precursors, *Hox* gene dysregulation and the eventual development of myeloid leukemia [4], [23], [54]. Transformation of MLL-AF9 fusion gene into mouse ES cells demonstrated that non-malignant expansion of myeloid precursors is the first stage of MLL-AF9-mediated leukemia followed by accumulation of malignant cells in bone marrow and other tissue [4]. The long latency *in vivo* and monoclonal nature of the leukemias suggest that the secondary genetic alterations are required [4], [55], [56]. Here, we provided additional evidence that dysregulation of 5′ Hoxa cluster is closely correlated with aberrant histone H3 modifications in *Hoxa9* locus. In AR1 cells the expression of *Hoxa5*, *a7* and *a9* was increased 20 to 40 fold higher than in AT1 cells, and in particular, *Hoxa10* expression was increased by 105 fold. In parallel with the dysregulation of 5′Hoxa cluster genes from a5 to a10, an enriched peak of trimethylated H3K4 was shifted from the 3′ to 5′ direction of the *Hoxa9* regulatory sequence, and repressive histone modifications were suppressed in aggressive AR1 cells as compared to AT1 cells (Fig.1). Our data suggest that the “progressive” epigenetic alterations occurred from AT1 to AR1 possibly contribute to the secondary mutations that are required for the development of leukemia in the MLL-AF9 murine model system [4], [22]. Taken together, our studies suggest a coordinated role for Cdx4 and the menin-MLL complex in modulating *Hoxa9* expression during MLL-associated myeloid transformations, while Cdx4 is no longer required in full-blown leukemia cells such as AR1 cells. Comparison of sequences in UniGene with Cdx4 and menin proteins between mouse and human complete genome indicates that these proteins are highly conserved with amino acid identity of 82.39% in Cdx4 and 95.78% in menin. The DNA sequence covering regulatory elements B to F at Mouse *Hoxa9* locus (Fig. 5A) possess 71% to 96% of DNA sequence identity to human *Hoxa9* locus. The identities of the element B and element E which contain Cdx4 binding sites are 77% and 92%, respectively. Therefore, our studies have significant biological implications in further deciphering the detailed mechanisms whereby *Hox* genes are regulated to control hematopoiesis and leukemogenesis as well as devising novel therapeutic interventions by targeting the menin pathway in humans.

## Materials and Methods

### Cell lines and cell culture

The AT1 cell line was generated from the murine bone marrow cells of *Men1^l/l^*, *Cre-ER* mice by introduction with retrovirus expressing MLL-AF9 fusion protein, as we previously described [13]. Similarly, the other MLL-AF9 cell line (AR1) was generated by transducing MLL-AF9 fusion protein into the bone marrow cells of *Men1^l/l^* mice. AT1 cells and AR1 cells were cultured in modified IMDM [Iscove's Modified Dulbecco's medium (StemCell Technologies Inc, Vancouver, BC) containing 200 mM non-essential amino acids, 200 mM L-glutamine, 10 U/ml penicillin and 10 µg/ml Streptomycin supplemented with 15% fetal bovine serum (FBS) (Harlan Bioproducts for Science, Indianapolis, IN) and IL3].

### Retroviral constructs and infection

Retroviral MSCV-*Cdx4*-ires-GFP and MSCV-ires-GFP vectors were kindly provided by George Daley [16]. The viruses were packaged to infect AT1 cells and AR1 cells as previously described [13]. The retrovirally transduced cells by MSCV-*Cdx4*-GFP and MSCV-GFP virus were highly purified on the expression of GFP using MoFlo Cell Sorter (Dako Cytomation).

### Quantitative Gene Expression Analysis

Total RNA was prepared with Trizol reagent, and cDNA was synthesized with random hexamers and Superscript II (Invitrogen). Taqman PCR reactions were performed with 1–2 µl of cDNA on a 7500 Fast Real-Time PCR System (Applied Biosystems) following the manufacturer's instructions. Primers and probes labeled at the 5′ end with FAM (MGB TaqMan Probe) were listed in the Supporting Information, Data S2. GAPDH (Mm99999915) and/or β-actin (Mm00607939) were used as internal controls for normalization. Data was analyzed using the comparative C_T_ method following the manual described (Applied Biosystem). The average C_T_ value from three independent assays was listed in the Supporting Information, Data S2.

### Luciferase assays

DNA fragments (regulatory elements) from the promoter and 5′ UTR regions were obtained by PCR using mouse tail genomic DNA as the template and cloned into the pGL3-basic vector (Promega) at *Kpn*-*BglII* restriction sites. All constructs were validated by restriction digestions and sequencing analyses. Co-transfections of 500 ng luciferase reporters (pA, pB, pC, pD, pE, pF or pGL3 empty vector) and 1 µg of expression vectors were performed in 12 well plates using Lipofectamine 2000 (Invitrogen) with 50 ng of pTK-Renilla (Promega) as an internal control. The luciferase activities were measured on TR717 Microplate Luminometer (Tropix, PE Applied Biosystem) using the Dual-luciferase assay kit (Promega). The following expression vectors were utilized: pSG5-*Cdx4* and pcDNA3-*Menin*, which were generated by inserting the mouse Cdx4 cDNA into pSG5 [36] and the human menin cDNA into pcDNA3 [57] respectively. The empty pcDNA3 was used as a vector control to normalize the total DNA for transfection.

### Chromatin Immunoprecipitation (ChIP) analysis

Chromatin immunoprecipitation assays were performed using the Chromatin Immunoprecipitation Assay Kit (Upstate, Lake Pacid, NY) and specific antibodies (See Supporting Information S1). As control for ChIP assays and for antibody specificity, equal amounts (5 µg) of IgG and antibodies were used in parallel reactions. Using Taqman Real-time PCR (RT-PCR), the immunoprecipitated DNA samples were quantified relative to input DNA and calculated based on the formula: % total_sample_ = [2^(ΔCTinput−ΔCTsample)^×% total_input_] [58]. To reduce background, ΔCT value is determined by subtracting the average CT_IgG_ value from the average CT_input_ value or CT_sample_ value. Primer and probe sequences were included in Supporting Information, Data S2.

### Electrophoretic mobility shift assay (EMSA)

EMSA was carried out as previously described [36] with the following modifications. The coding region of *Cdx4* was amplified from the pSG5-*Cdx4* vector using primers (Cdx4F2, cacc gtc acc atg tat gga atc tgc and Cdx4R2, ttc aga aac tat gac ctg ctg tat c). The PCR product was cloned into the pCR4-TOPO vector (Invitrogen). The resultant plasmid, pCR4-Cdx4, was sequenced and restriction digested to determine its insert orientation. The TnT3 Quick coupled transcription-translation system (Promega) was used to produce Cdx4 protein *in vitro*, while the TnT7 Quick coupled transcription-translation system (Promega) was used to produce Cdx4-R as a control from the same plasmid (pCR4-Cdx4). The *in vitro* translated products were immunoblotted with anti-Cdx4 antibody. Six wild type sequences of double-stranded end-labeled oliogonucleotides covering the following regions were tested for Cdx4 and Cdx4-R protein binding by EMSA: probe1, probe 2 and probe 3 located within element B, probe 4 and probe 5 within element D and probe 6 within element E.

### Western blotting

Whole-cell extracts were prepared followed by protein concentration determination. Equivalent amounts of protein from each sample were then subjected to SDS-polyacrylamide gel electrophoresis (SDS-PAGE) on a 10% gel, transferred to nitrocellulose, and processed for immunoblotting with the indicated antibodies [59].

## Supporting Information

Data S1 (0.03 MB DOC)

Data S2 (0.06 MB DOC)

Figure S1 Characterization of AT1 and AR1 cells. (A) AT1 and AR1 cells were seeded in 6-well plate in triplicate, 1×105 cells in each well. AR1 cells grow much faster than AT1 cells. (B) The expression profile of 5′Hoxa cluster genes in AT1 and AR1 cells was determined by quantitative real time RT-PCR analysis. Relative to AT1 cells, AR1 cells expressed significantly higher expression of Hoxa5, a7, a9 and a10, especially Hoxa10 with 105 fold of increase. (C) AT1 cells were predominately myeloblast morphology with a mixed population of a few differentiated myelomonocytes and myeloneutrophils, whereas, AR1 cells retained immature myelobalsts. Cells were stained with Giemsa and observed under light microscopy (Leisca, original magnification, ×40). Myeloblasts were morphologically defined by their large size, granular appearance and high nucleus-cytoplasm ratio [4], [23].
(12.81 MB TIF)

Figure S2 Identification of functional cis-elements at Hoxa9 regulatory region. (A) Diagram of Hoxa9 locus, showing locations of regulatory elements for luciferase assays. The regulatory elements were cloned into pGL3-basic vector to generate various reporter genes. (B) Luciferase assays were conducted in menin-expressing MEF26 cells. The luciferase activity was calculated relative to the empty vector control (pGL3-basic).
(4.74 MB TIF)
