# Supplementary material for: Cdx4 and Menin Co-Regulate Hoxa9 Expression in Hematopoietic Cells
Source: PLoS One. 2006 Dec 20;1(1):e47. doi: 10.1371/journal.pone.0000047 (PMC1762371; doi:10.1371/journal.pone.0000047)
Supplement: Data S1 — (0.03 MB DOC) [file pone.0000047.s001.doc]

**Supporting Data S1**

**Figure S1.** **Characterization of AT1 and AR1 cells.** (A) AT1 and AR1 cells were seeded in 6-well plate in triplicate, 1x10^5^ cells in each well. AR1 cells grow much faster than AT1 cells. (B) The expression profile of 5’Hoxa cluster genes in AT1 and AR1 cells was determined by quantitative real time RT-PCR analysis. Relative to AT1 cells, AR1 cells expressed significantly higher expression of *Hoxa5*, *a7*, *a9* and *a10*, especially *Hoxa10* with 105 fold of increase. (C) AT1 cells were predominately myeloblast morphology with a mixed population of a few differentiated myelomonocytes and myeloneutrophils, whereas, AR1 cells retained immature myelobalsts. Cells were stained with Giemsa and observed under light microscopy (Leisca, original magnification, x40). Myeloblasts were morphologically defined by their large size, granular appearance and high nucleus-cytoplasm ratio [4,23].

**Figure S2. Identification of functional cis-elements at *Hoxa9* regulatory region.** (A) Diagram of *Hoxa9* locus, showing locations of regulatory elements for luciferase assays. The regulatory elements were cloned into pGL3-basic vector to generate various reporter genes. (B) Luciferase assays were conducted in menin-expressing MEF26 cells. The luciferase activity was calculated relative to the empty vector control (pGL3-basic).
