# Supplementary material for: Cdx4 and Menin Co-Regulate Hoxa9 Expression in Hematopoietic Cells
Source: PLoS One. 2006 Dec 20;1(1):e47. doi: 10.1371/journal.pone.0000047 (PMC1762371; doi:10.1371/journal.pone.0000047)
Supplement: Data S2 — (0.06 MB DOC) [file pone.0000047.s002.doc]

**Supporting Data S2**

**1. Primers and probes used for qChIP assay**

**Amplicon 1**

Primer 1F: AAGGTGTGCATAAGGGAAGCTGGT;

Primer 1R: TGGATCTGGCAAAGTGTTTCCTGC

Probe 1: AGACAAGGCCTCGGTTACGATCACG

**Amplicon 2**

Primer 2F: TCCCTCCAACTCTTCTCTGCCATT

Primer 2R: ATGCAACCACACAACACCTCATGC

Probe2: TGCATCCGGGTACTGGGCTTATTTCA

**Amplicon 3**

Primer 3F: AGATGGAAATGTCTCGGGTCAGCA

Primer 3R: AGGCCACAGCCACTGTTGTTAATG

Probe3: TGCTTTGTGTTCCAGATTGGGATGGG

**Amplicon 4**

Primer 4F: AACGAATCTGTTGGTCGCTCCTGA

Primer 4R: GCAGCCAAATCGCATTCTCACTCT

Probe 4: ACCAGCCTGTGTGGCTTCCGAAACAATA

**2. Oligo probes used for EMSA**

probe 1: caaaagaaagcagaaataaaaacctgta

probe 2: caatatctcct**tttatg**aattttccccctttt

probe 3: ttaaaagtattttaaatt**aataaa**tattaat

probe 4: cacaaaattcacacgtgaatttaattacac

probe 5: aagattctaaggggtgaaaaataacatcctccatttg

probe 6: TGCTTT**TTTATG**GCTTCAATTATTGTCTAAT**TTTATG**TGA

probe 2 (mut1) CAATATCTCCTTccAgGAATTTTCCCCCTTTT

probe 2 (mut2) CAATATCTCCTTaaAcGAATTTTCCCCCTTTT

probe 6(mut6) TGCTTTTccAgGGCTTCAATTATTGTCTAATTaaAcGTGA

**3. Antibodies used for ChIP assays**:

Control IgG (normal rabbit IgG, Santa Cruz Biotechnology, sc2027), anti-menin (Bethyl Lab, A300-105A), anti-H3K4M3 (abcam, ab8580), anti-H3K4M2 (abcam ab7766), anti-H3K4M1 (abcam, ab8895), anti-H3K9M3 (abcam, ab8898), anti-K9M2 (abcam, ab7312), anti-H3K9M1 (abcam, ab9045), anti H3K27M2 (abcam, ab24684), anti-H3K27M3 (abcam, ab6002), anti-H3K9Ace (abcam, ab4441), anti-H4K16Ace (abcam,ab1762), anti-SUZ12 (abcam, ab12201), Anti-EZH2 (Upstate, 07-400), anti-Cdx4 (Aviva System Biology, ARP32765-P100; ABGENT, AP6132a)

**4. Quantitative gene expression analysis using quantitative real time RT-PCR (qPCR)**

**qPCR 1 (Fig. S**1B, Fig. 3C)

| Gene | MGB TaqMan Probe | Average ΔC_T_ | | |
| --- | --- | --- | --- | --- |
|  |  | *Men1*^l/l^ | *Men1*^Δ/Δ^ | AR1 |
| *Men1* | Mm00484963_m1 | 6.516±0.345 | 10.124±1.019 | 7.712±0.16 |
| *Hoxa1* | Mm00439359_m1 | undetectable | undetectable | 10.685±0.4 |
| *Hoxa5* | Mm00439362_m1 | 12.995±0.133 | 14.23±0.212 | 7.518±0.173 |
| *Hoxa7* | Mm00657963_m1 | 9.893±0.032 | 11.985±0.499 | 5.31±0.389 |
| *Hoxa9* | Mm01332950_g1 | 11.671±0.19 | 13.664±0.236 | 6.304±0.118 |
| *Hoxa10* | Mm00433966_m1 | 13.585±0.206 | 15.201±0.113 | 6.863±0.038 |
| *Hoxa13* | Mm00433966_m1 | undetectable | undetectable | 18.022±0.986 |

qPCR 2 (Fig. 4A)

| Gene | MGB TaqMan Probe | Average ΔC_T_ | | |
| --- | --- | --- | --- | --- |
|  |  | AT1 | AR1 | MEF |
| *Men1* | Mm00484963_m1 | 7.885±0.122 | 8.704±0.083 | 7.712±0.16 |
| *MLL* | Mm01179213_g1 | 8.572± 0.2 | 8.431±0.052 | 9.07±0.118 |
| *Cdx4* | Mm00432452_m1 | 17.838±0.114 | 21.525±0.016 | undetectable |
| *Hoxa9* | Mm01332950_g1 | 10.284±0.194 | 3.723±0.262 | 11.448±0.043 |

**qPCR** 3 (Fig. 4B, 4C)

| Gene | MGB TaqMan Probe | Average ΔC_T_ | | | |
| --- | --- | --- | --- | --- | --- |
|  |  | AT1+GFP | AT1+Cdx4 | AR1+GFP | AR1+Cdx4 |
| *Men1* | Mm00484963_m1 | 9.936±0.52 | 9.825±0.908 | 9.954±0.82 | 9.676±0.394 |
| *Cdx4* | Mm00432452_m1 | 17.44±0.456 | 3.475±0.632 | 19.855±0.752 | 3.484±0.532 |
| *Hoxa5* | Mm00439362_m1 | 9.192±0.425 | 7.814±0.145 | 9.229±0.272 | 8.542±0.192 |
| *Hoxa7* | Mm00657963_m1 | 8.172±0.413 | 7.208±0.259 | 8.529±0.296 | 8.027±0.466 |
| *Hoxa9* | Mm01332950_g1 | 7.129±0.411 | 6.131±0.335 | 6.662±0.271 | 6.518±0.4 |
| *Hoxa10* | Mm00433966_m1 | 5.849±0.289 | 4.617±0.286 | 5.761±0.101 | 5.278±0.472 |
